# Engineering a filamentous fungus for l-rhamnose extraction

**DOI:** 10.1186/s13568-016-0198-8

**Published:** 2016-03-31

**Authors:** Joosu Kuivanen, Peter Richard

**Affiliations:** VTT Technical Research Centre of Finland Ltd., PO Box 1000, 02044 VTT Espoo, Finland

**Keywords:** l-rhamnose, Rhamogalacturonan, Pectin, Citrus peel, *Aspergillus niger*, Metabolic engineering, Consolidated bioprocess

## Abstract

l-Rhamnose is a high value rare sugar that is used as such or after chemical conversions. It is enriched in several biomass fractions such as the pectic polysaccharides rhamnogalacturonan I and II and in naringin, hesperidin, rutin, quercitrin and ulvan. We engineered the filamentous fungus *Aspergillus niger* to not consume l-rhamnose, while it is still able to produce the enzymes for the hydrolysis of l-rhamnose rich biomass. As a result we present a strain that can be used for the extraction of l-rhamnose in a consolidated process. In the process the biomass is hydrolysed to the monomeric sugars which are consumed by the fungus leaving the l-rhamnose.

## Introduction

l-Rhamnose is a 6-deoxy sugar that is commercially used by different industries as a precursor compound or as such. In the biomass, l-rhamnose occurs typically as bound to other compounds, such as in glycosides and polysaccharides. In plants, it is found as a sugar unit in glycosides, such as naringin and hesperidin in citrus fruits or rutin and quercitrin in oak bark (Linhardt et al. 1989; Garg et al. 2001). l-Rhamnose occurs also in the pectic polysaccharides rhmanogalacturonan (RG) I and II in the plant cell wall in which it is found in the repeating backbone disaccharide with d-galacturonate (RGI) or from the side chains (RGII) (Mohnen 2008). Another l-rhamnose containing cell wall polysaccharide is ulvan—the predominant component in the cell wall in some green algae species (Lahaye and Robic 2007). l-Rhamnose is a major constituent in ulvan comprising between 16.8 and 45 % of the ulvan constituents (Lahaye and Robic 2007). In addition to plant and algal biomass, some bacteria produce rhamnolipids, in which l-rhamnose occurs as glycoside head group attached to a fatty acid tail (Lang and Wullbrandt 1999), and cell wall exopolysaccharides containing l-rhamnose (Bryan et al. 1986).

Several microbial species are capable of utilizing l-rhamnose as carbon and energy source. In the first stage l-rhamnose monomers are enzymatically hydrolysed from biomass glycosides and polysaccharides by excreted α-l-rhamnosidases. In bacteria, monomeric l-rhamnose is catabolized through a pathway containing isomerization and phosphorylation as first steps (Takagi and Sawada 1964a, b). In the fungal pathway, l-rhamnose is first oxidized to l-rhamnono-1,4-lactone which is then hydrolyzed to l-rhamnonate, dehydrated to l-2-keto-3-deoxy-rhamnonate and split to pyruvate and l-lactaldehyde (Twerdochlib et al. 1994). The enzymes in the fungal pathway are l-rhamnose-1-dehydrogenase (Koivistoinen et al. 2008), l-rhamnono-1,4-lactone lactonase, l-rhamnonate dehydratase and l-2-keto-3-deoxy-rhamnonate aldolase encoding by the genes *RHA1*, *LRA2*, *LRA3* and *LRA4*, respectively (Watanabe et al. 2008; Koivistoinen et al. 2012).

l-Rhamnose as a free sugar is commercially utilized in food, beverage, cosmetic and pharmaceutical industries. For example, it is used as a precursor in furaneol synthesis which is a strawberry aroma compound supplemented to food and soft drinks (Illmann et al. 2009). Another example of application areas is cosmetics where l-rhamnose is used as an anti-aging agent (Robert et al. 2012). Traditionally commercial l-rhamnose is extracted from vegetable matter containing l-rhamnose in glycosides (Linhardt et al. 1989) or purified from lignocellulosic biomass hydrolysates (Saari et al. 2010). l-Rhamnose is released by chemical hydrolysis following extraction from the other constituents. Due to the laborious manufacturing method l-rhamnose is considered as an expensive fine chemical (Linhardt et al. 1989). Alternative raw materials for the chemical hydrolysis for l-rhamnose production include algal biomass (Takemura et al. 1988) and bacterial rhamnolipids (Daniels et al. 1990). In addition, a biotechnological manufacturing process including enzymatic hydrolysis of l-rhamnose from biomass glycosides or pectin by added enzymes and a fermentation step with yeast for removing other sugars resulting from hydrolysis has been described in patent literature (Cheetham 1989).

In the present study, we have developed a one-step biotechnological process for the l-rhamnose extraction using an engineered strain of the filamentous fungus *Aspergillus niger*. In the engineered strain the catabolic l-rhamnose pathway is disrupted and l-rhamnose catabolism blocked by gene deletions. The resulting strain is still capable of hydrolyzing l-rhamnose monomers from various biomass compounds. The strain is able to valorize grape fruit peel, an agricultural waste, to l-rhamnose in a consolidated bioprocess without any biomass pretreatment steps.

## Methods

### Strain construction

**Fig. 1:**
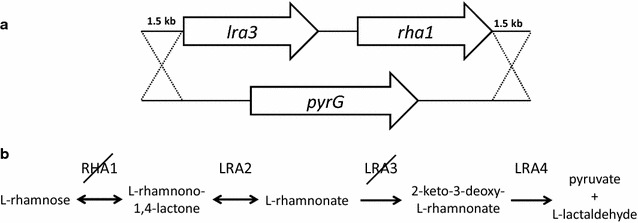
Deletion of *rha1* and *lra3* genes: (**a**) Deletion cassette for disruption of the catabolic l-rhamnose pathway in *A. niger* (**b**). The enzymes in the pathway are RHA1 (l-rhamnose dehydrogenase), LRA2 (l-rhamnono-1,4-lactonase), LRA3 (l-rhamnonate dehydratase) and LRA4 (2-keto-3-deoxy-l-rhamnonate aldolase)

The *A. niger* strain ATCC 1015 (CBS 113.46) was used as a wild type. All the DNA construction steps were carried out in *Escherichia coli* TOP10 cells. For disrupting the catabolic l-rhamnose pathway in *A. niger*, the clustered genes coding for l-rhamnose-1-dehydrogenase and l-rhamnonate dehydratase (*rha1* and *lra3*) were deleted with a single deletion cassette (Fig. 1a). The deletion cassettes contained the *pyrG* gene for selection and 1.5 kb flanks targeted to the 5′ region of the *lra3* and the 3′ region of the *rha1* gene. The 5′ and 3′ flanking region were amplified from ATCC 1015 genomic DNA by using Phusion DNA polymerase (Thermo Scientific). The primer pair for the 5′ flank (TATA*CTCGAG*GCGTTATTCG-TCGACCGTGG/TATA*GAATTC*TCCCGGGGAAACTGGAGCAT) contained *Xho*I and *Eco*RI sites and the primer pair for the 3′ flank (TATA*GCGGCCGC*GTGGTCA-ACCCACCCCCTTA/TATA*CCGCGG*CCTCGTTCAGGATAAGCCGA) *Not*I and *Sac*II sites. The resulting flanks were ligated into the pRS426 derived plasmid containing *A. nigerpyrG* gene in *Xma*I site. The 5′ flank was ligated to the front (*Xho*I/*Eco*RI) and 3′ flank behind (*Not*I/*Sac*II) the *pyrG* gene, respectively, using suitable restriction enzymes (Thermo Scientific) and T4 DNA ligase (NEB). The resulting deletion cassette was linearized with *Xho*I and *Sac*II before *A. niger* was transformed.

The *A. niger* ATCC 1015 ∆*pyrG* strain (Mojzita et al. 2010) was used as platform for *rha1* and *lra3* deletions. The deletion cassette was transformed using the protoplast transformation method. Transformants were screened for growth in the absence of uracil. Integration of the cassette and *rha1* and *lra3* double deletion was verified by colony PCR using Phire direct PCR kit (Thermo Scientific) and the primers AGGGGAAAGTCTGCCGG and ATGGCTCTTCCAGACGGTAG. The resulting *A. niger* strain ∆*rha1*∆*lra3* (*pyrG*∆, *rha1*::*pyrG*, *lra3*::*pyrG*) was used in the cultivations for l-rhamnose extraction.

### Media and culture conditions

Luria Broth (LB) culture medium supplemented with 100 µg ml^−1^ of ampicillin and cultural conditions of 37 °C and 250 rpm were used for *E. coli* cultures.

*Aspergillus niger* spores were generated on potato-dextrose plates and ~10^8^ spores were inoculated into 50 ml of YP medium (10 g yeast extract l^−1^, 20 g peptone l^−1^) containing 30 g gelatin l^−1^ for pre-cultivation. Mycelia were pre-grown in 250-ml Erlenmeyer flasks by incubating overnight at 28 °C, 200 rpm and harvested by vacuum filtration, rinsed with sterile water and weighted. *Aspergillus nidulans* defined minimal medium (Barratt et al. 1965), containing (in g l^−1^) 6 NaNO_3_, 0.52 KCl, 0.52 MgCl_2_ and 1.52 KH_2_PO_4_ was used in all the *A. niger* agar plates and liquid cultivations. In the transformation, the minimal medium was supplemented with 1.2 M d-sorbitol, 10 g l^−1^ of d-glucose and 20 g l^−1^ of agar and the pH was adjusted to 6.5. For testing the phenotype, the minimal medium was supplemented 20 g l^−1^ agar and 9 g l^−1^l-rhamnose, or in the liquid cultivations with 22 g l^−1^l-rhamnose. In the cultivations for investigating l-rhamnose extraction, the minimal medium was supplemented alternatively with 20 g l^−1^ naringin (Sigma), 10 g l^−1^ RG (Megazyme, extracted from soy bean), or 122 g l^−1^ ground grape fruit peel (dry mass basis). The pH was adjusted to 3. The grape fruit peels were generated from pink grape fruits (*Citrus paradisi*) that were obtained from a local grocery. Peels were ground in a blender and autoclaved without any additional washing or drying. All the liquid cultivations were carried out in 250 ml Erlenmeyer flasks and in 50 ml liquid volume and were inoculated with ~1 g l^−1^ of pre-grown mycelia (dry mass basis); l-rhamnose plates with 5 × 10^6^ spores.

### Chemical analyses

Samples of 2 ml were removed from the liquid cultivations at intervals and mycelium was separated from the supernatant by centrifugation. The concentrations of l-rhamnose, other sugars and sugar acids were determined by HPLC using a Fast Acid Analysis Column (100 mm × 7.8 mm, BioRad Laboratories, Hercules, CA) linked to an Aminex HPX-87H organic acid analysis column (300 × 7.8 mm, BioRad Laboratories) with 5.0 mM H_2_SO_4_ as eluent and a flow rate of 0.5 ml min^−1^. The column was maintained at 55 °C. Peaks were detected using a Waters 410 differential refractometer and a Waters 2487 dual wavelength UV (210 nm) detector.

## Results

**Fig. 2:**
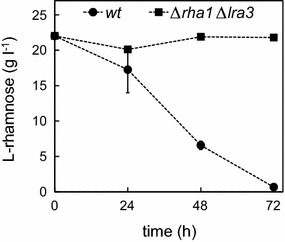
Flask cultivations on l-rhamnose with *A. niger* wild type (*circles*) and *∆rha1∆lra3* (*squares*). *Error bars* represent ± standard deviation (n = 3) and where not visible, are smaller than the symbol

The filamentous fungus *A. niger* is capable of catabolizing l-rhamnose and the orthologous genes encoding the fungal catabolic pathway are found from the *A. niger* genome where they are organized in a cluster (Koivistoinen et al. 2012). With the exception of the regulatory protein RhaR (Gruben et al. 2014) and the l-rhamnonate dehydratase LRA3 (Motter et al. 2014), none of the putative l-rhamnose pathway genes or enzymes in *A. niger* have been characterized nor was the function demonstrated. In this communication, the clustered genes encoding a putative l-rhamnose-1-dehydrogenase (JGI protein ID 124656) and the recently characterized l-rhamnonate dehydratase (JGI protein ID 1081712), *rha1* and *lra3*, respectively, were deleted with a single gene deletion cassette (Fig. 1). The resulting strain ∆*rha1*∆*lra3* was tested for l-rhamnose catabolism on agar plates containing l-rhamnose and in liquid cultivations in which l-rhamnose was added as only carbon source. The wild type strain grew on l-rhamnose plates (data not shown) and consumed all the l-rhamnose in the liquid cultivations (Fig. 2). The strain ∆*rha1*∆*lra3* did not show any growth on l-rhamnose (data not shown) nor l-rhamnose consumption under these conditions (Fig. 2) indicating that the genes deleted were indeed required for l-rhamnose catabolism in *A. niger*. In addition, we have tested the ∆*rha1*∆*lra3* strain for l-rhamnose dehydrogenase activity with NAD^+^ resulting in no detectable activity (data not shown). Thus the gene *rha1* most likely encode a functional l-rhamnose-1-dehydrogenase.

**Fig. 3:**
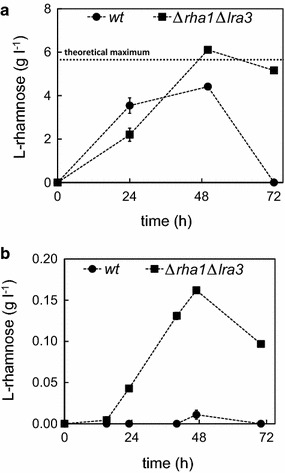
Flask cultivations with *A. niger* wild type (*circles*) and *∆rha1∆lra3* (*squares*) on naringin (**a**) and RGI from soy (**b**). *Error bars* represent ± standard deviation (n = 3) and where not visible, are smaller than the symbol. The theoretical maximum in the **a** represents the l-rhamnose amount that is bound in the naringin amount used

As a next step, two different l-rhamnose containing biomass components—the flavonoid naringin and the pectic polysaccharide RG—were tested as raw materials for l-rhamnose extraction using the ∆*rha1*∆*lra3* strain. Pure naringin and RGs extracted from soy beans were used as raw material for l-rhamnose extraction in liquid cultivations with the wild type and ∆*rha1*∆*lra3* strains (Fig. 3). In the case of naringin, a concentration of more than 6 g l^−1^ of free l-rhamnose was achieved within 48 h with ∆*rha1*∆*lra3* (Fig. 3a). The initial naringin concentration (20 g l^−1^ = 34.5 mM) contains around 6 g l^−1^ of l-rhamnose. Thus the observed l-rhamnose corresponds to the total l-rhamnose content in the used naringin. A small l-rhamnose loss was observed in the cultivation between 48 and 72 h. The wild type strain did accumulate some free l-rhamnose, however, the concentrations were lower and all the l-rhamnose was consumed within 72 h (Fig. 3a). In the cultivations with RG and with the ∆*rha1*∆*lra3* strain, the l-rhamnose concentration of 0.16 g l^−1^ was observed after 47 h. According to the manufacturer, the l-rhamnose content in the used RG was 13 %. Thus the highest achievable concentration of free l-rhamnose in the used RG-medium (10 g l^−1^) should be 1.3 g l^−1^. As well as with naringin, a small loss of l-rhamnose was observed in the end of cultivations with ∆*rha1*∆*lra3*. With the wild type strain, only residual l-rhamnose concentrations were observed in the cultivation with RG (Fig. 3b).

The residue from grape fruit processing is a waste biomass containing both naringin and pectin. For that reason, grape fruit peels were tested as a potential raw material for l-rhamnose extraction in a consolidated bioprocess in liquid cultivations using the engineered strain. Similar to the earlier experiments with naringin and RG, the ∆*rha1*∆*lra3* strain did accumulate l-rhamnose from grape fruit peels (Table 1). The l-rhamnose concentration of 1.73 g l^−1^ was observed after 50 h with the engineered strain while the wild type strain had consumed all the l-rhamnose. Another main compound observed in the cultivations was d-galacturonate that is the main monomer in pectin. In the cultivations with the wild type strain, all the l-rhamnose was consumed prior to d-galacturonate. With the ∆*rha1*∆*lra3* strain, a slow consumption of l-rhamnose was observed again but only after the consumption of d-galacturonate (data not shown).

**Table 1:** Flask cultivations supplemented with 122 g l^–1^ (dry mass) of grape fruit peels with *A. niger* wild type and *∆rha1∆lra3* after 24 and 50 h error represent ± standard deviation (n = 3)

Strain	l-rhamnose (g l^–1^)		d-galacturonate (g l^–1^)	
	24 h	50 h	24 h	50 h
*∆rha1∆lra3*	0.40 ± 0.35	1.73 ± 0.04	0.49 ± 0.10	1.36 ± 0.28
*wt*	0.77 ± 0.04	0	0.48 ± 0.04	1.25 ± 0.10

## Discussion

The aim of the present study was to develop a single step biotechnological process for the extraction of l-rhamnose from biomass. l-Rhamnose is a high value sugar that is used in different applications. The catabolic l-rhamnose pathway was disrupted from the filamentous fungus *A. niger*. The engineered strain was still capable of hydrolysing l-rhamnose containing biomass and it was used for the extraction of l-rhamnose from naringin, RG and grape fruit peel in a consolidated process.

Naringin is a flavanone (a flavonoid with a disaccharide unit at position seven) consisting of naringenin and a disaccharide unit of d-glucose and l-rhamnose. It occurs naturally in citrus fruits and is especially abundant in grape fruits in which it is responsible for the bitter taste (Puri 2012). Naringinase is the enzyme activity including β-glucosidase and α-rhamnosidase activities resulting in hydrolysis of the disaccharide unit of naringin (Puri 2012). Naringinases are produced by several fungal species including *A. niger* (Puri 2012) meaning that these fungi should be capable of hydrolyzing naringin in vivo as well. In the present study, it was demonstrated that the engineered ∆*rha1*∆*lra3* strain can indeed hydrolyze naringin resulting in l-rhamnose accumulation in the medium. As a result, an l-rhamnose yield close to the maximum theoretical yield was achieved from naringin.

RGI and RGII are pectic polysaccharides accounting for about 20–35 % and 10 %, respectively, of the pectin (Mohnen 2008). The backbone in RGI is composed of repeating rhamnose-d-galacturonate disaccharide unit while RGII contains rhamnose in its side chains. As well as some other pectin types, RGI and II contain complex side chains and methylated and acetylated backbone components (Mohnen 2008). Thus a mix of different pectic enzymes is needed for pectin hydrolysis. *Aspergillus niger* is known to be efficient in pectin hydrolysis and is also capable of hydrolyzing pectin from citrus peels in a consolidated bioprocess (Kuivanen et al. 2014). However, a lower l-rhamnose yield was achieved from RG when compared to naringin. This means that either the RG hydrolysis was incomplete or released l-rhamnose was already consumed at the early state of the cultivation. This was however not observed in the naringin cultivations.

l-Rhamnose accumulation by the engineered fungus was also observed in a consolidated process from grape fruit peels which contain both naringin and RG. Grape fruits are known to be rich in naringin. The results from the naringin and RG cultivations with the engineered strain support more efficient l-rhamnose release from naringin than from RG. Thus it is likely that observed l-rhamnose in the grape fruit peel cultivations is mainly derived from naringin. This hypothesis is also supported by the fact that other citrus fruit peels that we have tested with the engineered strain resulted in lower l-rhamnose concentrations.

In the cultivations where l-rhamnose was added as a sole carbon source, no rhamnose consumption was observed by the ∆*rha1*∆*lra3* strain. However, when additional carbon sources were available from naringin, RG or grape fruit peel, slow l-rhamnose disappearance was observed. This may be due to unspecific enzyme activities from other catabolic sugar and sugar acid pathways, such as d-galacturonate or d-xylose pathways resulting in slow oxidation or reduction, and further catabolism of l-rhamnose. Nevertheless, with a careful process optimization and correct timing of the l-rhamnose harvest from the cultivations, the unwanted loss can be eliminated.

In addition to naringin, RG and grape fruit peel, we have tested the l-rhamnose extraction from the algal polysaccharide ulvan with the engineered strain. In ulvan, a sulfated aldobiouronic acid, a disaccharide of C-3 sulfated rhamnose and d-glucuronic acid, is the most abundant repeating structural unit. Ulvanolytic enzymes, such as ulvan-lyase cleaving the linkage between rhamnose 3-sulfate and glucuronic acid are required in ulvan degradation (Lahaye and Robic 2007; Hehemann et al. 2014). We did not observe l-rhamnose or any other ulvan constituents as free compounds in the cultivations indicating that the hydrolysis of ulvan is not efficient in *A. niger*.

In the present communication, we established a consolidated process for l-rhamnose isolation from naringin and grape fruit peel. The process is similar to the processes of l-galactonate (Kuivanen et al. 2014) and l-ascorbic acid (Kuivanen et al. 2015) production from the d-galacturonate fraction in orange peel. The microorganism is producing the enzymes for the hydrolysis of the biomass and is simultaneously removing most of the sugars except for the l-rhamnose in a single process. The biotechnological method described here may offer a greener alternative to the current chemical l-rhamnose extraction processes.
